# Insights into Flexible Bioinspired Fins for Unmanned Underwater Vehicle Systems through Deep Learning

**DOI:** 10.3390/biomimetics9070434

**Published:** 2024-07-17

**Authors:** Brian Zhou, Kamal Viswanath, Jason Geder, Alisha Sharma, Julian Lee

**Affiliations:** 1 Laboratories for Computational Physics and Fluid Dynamics, United States Naval Research Laboratory, Washington, DC 20375, USA; jason.d.geder.civ@us.navy.mil (J.G.); alisha.j.sharma.civ@us.navy.mil (A.S.); julian.lee@yale.edu (J.L.); 2 Harvard College, Cambridge, MA 02138, USA; 3 Department of Computer Science, University of Maryland, College Park, MD 20742, USA; 4 Yale College, New Haven, CT 06520, USA

**Keywords:** unmanned underwater vehicles, bio-inspired robotics, control system optimization, gait analysis, power-aware robotics, figure of merit, deep learning, inverse models

## Abstract

The last few decades have led to the rise of research focused on propulsion and control systems for bio-inspired unmanned underwater vehicles (UUVs), which provide more maneuverable alternatives to traditional UUVs in underwater missions. Recent work has explored the use of time-series neural network surrogate models to predict thrust and power from vehicle design and fin kinematics. We expand upon this work, creating new forward neural network models that encapsulate the effects of the material stiffness of the fin on its kinematic performance, thrust, and power, and are able to interpolate to the full spectrum of kinematic gaits for each material. Notably, we demonstrate through testing of holdout data that our developed forward models capture the thrust and power associated with each set of parameters with high resolution, enabling highly accurate predictions of previously unseen gaits and thrust and FOM gains through proper materials and kinematics selection. As propulsive efficiency is of utmost importance for flapping-fin UUVs in order to extend their range and endurance for essential operations, a non-dimensional figure of merit (FOM), derived from measures of propulsive efficiency, is used to evaluate different fin designs and kinematics and allow for comparison with other bio-inspired platforms. We use the developed FOM to analyze optimal gaits and compare the performance between different fin materials. The forward model demonstrates the ability to capture the highest thrust and FOM with good precision, which enables us to improve thrust generation by 83.89% and efficiency by 137.58% with proper fin stiffness and kinematics selection, allowing us to improve material selection for bio-inspired fin design.

## 1. Introduction

Autonomous and unmanned underwater vehicles (AUV/UUVs) have a variety of industrial and research applications, including exploration, mapping, and minesweeping. Historically, these operations have primarily been conducted with propeller-driven UUVs. Because propeller-driven UUVs lack maneuverability and, subsequently, resistance to turbulence, their operational domain is limited to relatively quiescent and deeper waters. Marine animals offer promising solutions to expanding the envelope of UUV operations because they swim with high propulsive efficiency and have high maneuverability in water [1,2,3,4,5,6,7], which motivates the replication of their fins and other appendages in robotic designs [8]. As such, recent decades have seen the rise of research in bio-inspired propulsion systems to fill the operational gap in littoral waters and create systems with greater agility and maneuverability compared to traditional propeller-based systems. These bio-inspired propulsion systems have additional benefits: (i) multiple fins allow for better maneuvering and motion stabilization compared to conventional propulsion systems, (ii) bio-inspired designs are easier to mask acoustic/hydrodynamic signatures, and (iii) bio-inspired fins have a lower environmental impact compared to screw propellers or turbines which may harm vegetation and marine life [9]. Fin designs inspired by a variety of animals have been studied, including dolphins [4,5], penguins [1], and snakes. Among these, fish-inspired fins have driven the majority of research due to the agility these species exhibit, outperforming the capabilities of traditional propulsion-based systems [7,10,11,12].

Replicating the performance of biological flapping fins with bio-inspired robotic systems requires design, testing, and optimization of different parameters. Previous studies have extensively examined and tested the effects that different parameters such as material properties, kinematics or fin gaits, and fin shape have on a flapping fin’s thrust output to better replicate and understand fish hydrodynamic performance [12,13,14,15,16,17,18,19]. While previous studies have studied the effects of various design and parameter choices on thrust and lift forces [12,13,14,15,16,17], less research has been conducted on the effects designs and flapping gaits have on power consumption, preventing major studies on overall propulsive efficiency. In the cases that evaluate the effects of various designs and gaits on power consumption, analysis often uses CFD simulations to study and model hydrodynamic power consumption [20], but this is ineffective for control system integration that requires simulation to create real-time predictions, be computationally inexpensive, and take into account power loss of integrated actuators; CFD simulations are unable to be run in real-time on unmanned systems, and they are often too computationally intensive to be run on the unmanned system, making them impractical for autonomous systems. While some research has also studied the effects of design choices on power consumption and gait efficiency [18,19], these studies present insights into which designs or shapes may be more efficient overall, not which individual movements with a certain design might be more efficient. Analyzing practical power draws of individual movements and building models will allow us to create better design and gait recommendations while considering propulsive efficiency and integrating power into a model-driven control system operating in real-time. This allows a control system to have different gait settings, such as a toggle-able setting optimizing for force output or gait efficiency, drastically increasing the possible mission duration for size-constrained littoral UUVs.

To address the shortcomings of previous fin modeling and control approaches, we propose a dual neural network model to embed a more comprehensive understanding of the relationship between gait and propulsion as well as gait and power consumption by predicting the power consumed and thrust generated from the time series of any given gait. As a result, the control system can not only generate gaits that meet a desired trajectory but also optimize for important performance measures such as rapid propulsion acceleration, a smooth motor transition, and greater energy efficiency. By providing insights into both the propulsion and the power consumption of any singular gait, we are able to develop a nondimensionalized approach to evaluating the efficiency of various kinematics or materials. This allows for direct comparison of the overall efficiency of various designs, aiding optimization in offline time by improving material insights and in online time by improving gait-to-gait comparisons.

This paper is organized into two main sections. The Section 2 outlines the design parameters for the fins and fin kinematics, as well as the details of the experimental setup for collecting force and power data. The Section 3 details the development of a neural network model which demonstrates precise and accurate prediction of thrust and power for a given set of fin kinematics, as well as a measure of propulsive efficiency defined by a derived figure of merit. While prior studies have emphasized the effects of designs and gait on propulsion [12,13,14,15,16,17], we resolve to expand modeling to the effects that fin designs and gaits have on power consumption and UUV efficiency. Previous research in power utilizes CFD simulations to study hydrodynamic power ineffective for use in a control system that requires accounting for the power loss of integrated actuators [20]. By training our deep learning model on actuator data, our model is able to understand the efficiency of each movement in live time while accounting for actuator loss. This is especially important for bio-inspired UUVs, which are typically small and lightweight, limiting the battery size and electronic compute power. Our approach advances live-time understandings of real-world power consumption and efficiency, while previous CFD approaches would be computationally intensive, unfeasible to obtain predictions in live time, and unable to predict power inefficiencies from actuators.

## 2. Materials and Methods

### 2.1. Experimental Setup

**Figure 1:**
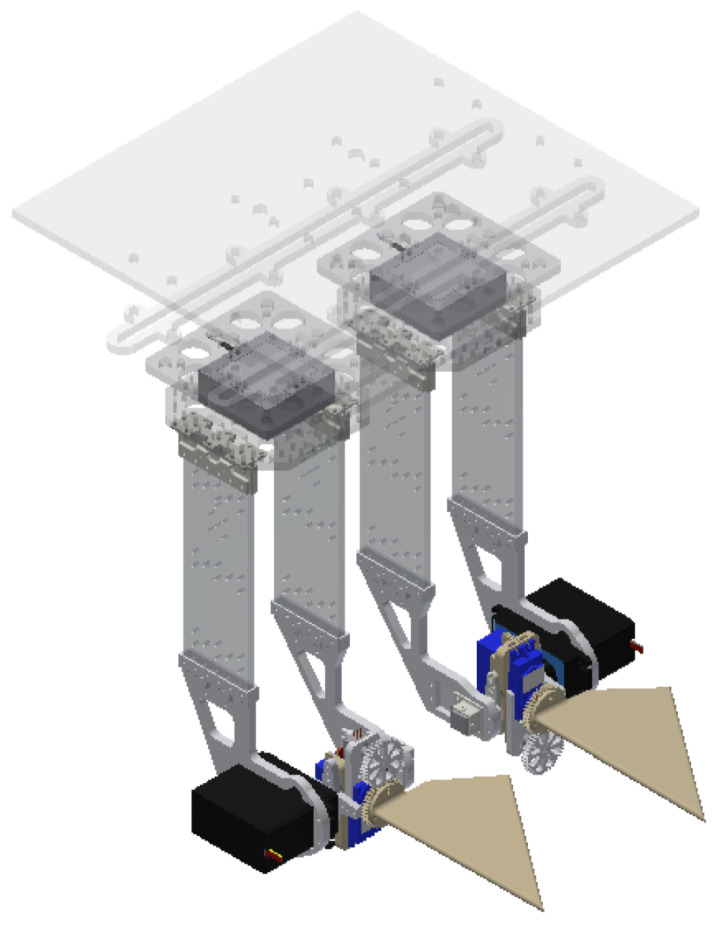
CAD design of tandem fins mounted on sensors and a control platform.

**Figure 2:**
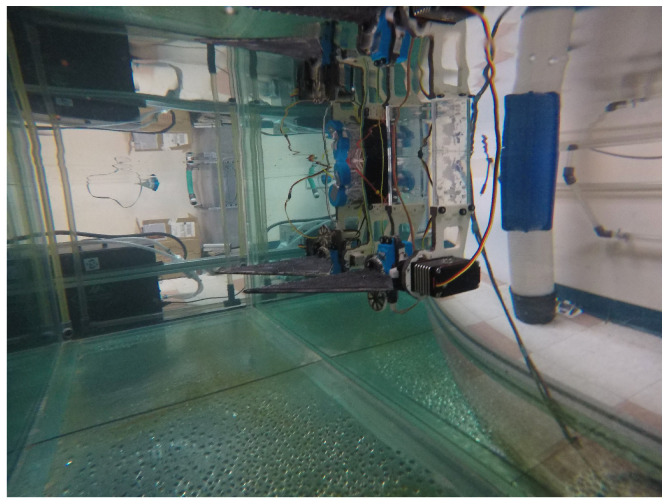
Tandem fin testing platform in the experimental test environment.

To train and evaluate both the forward and inverse models, experimental data were collected for a system of artificial pectoral fins, whose geometries are outlined in Sampath et al. [13], mounted in tandem in an underwater tank shown in Figure 1 and Figure 2. For this study, we were interested in the propulsive performance of the front fin, and studies that characterize the effects of flow on the rear fin will be considered in future work. As there are no upstream effects on the front fin [13], it is essentially treated as an isolated fin. Further, when spaced further apart, as may be the case with unmanned vehicles, flow interactions between tandem fins also become negligible for rear fin performance [17].

The control platform was mounted on a 2.41 × 0.76 × 0.76 m (length, width, height) glass tank. A microcontroller was programmed to define fin kinematics and control the fin actuators, and thrust and power data were collected for various programmed gait combinations. The relevant kinematics parameters are defined in Table 1 and Table 4. Calibrated potentiometers (TT Electronics P260) measure the stroke and pitch angles over time. Although only the static kinematic values (*f*, δ, Φ, and Θ) define a unique gait, measurement of dynamic kinematic values (ϕ and θ) is useful for more accurate forward model training and interpolation.

### 2.2. Parameters and Outputs

**Figure 3:**
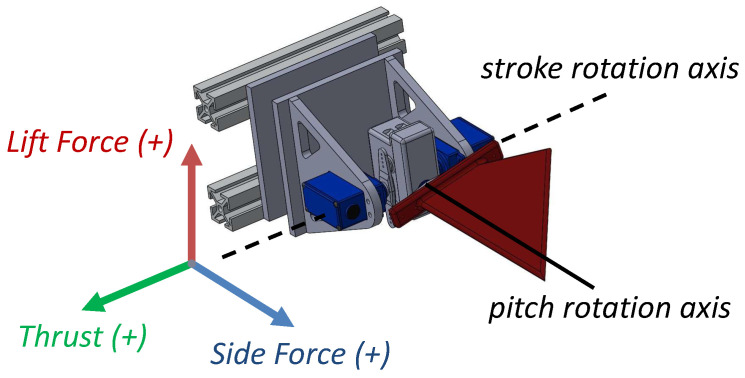
CAD design of a single flapping fin propulsor with coordinate reference frames.

A singular gait is comprised of four kinematic parameters: stroke phase offset (δ), stroke amplitude (Φ), pitch amplitude (Θ), and flapping frequency (*f*). For each unique gait, we collected experimental data on the forces generated in the *x*, *y*, and *z* axes, the current drawn by each motor, and the circuit voltage for different fin gaits. These parameters are defined in Table 2, and force vectors are illustrated in Figure 3. Load cells (Interface 3A60A) measure the generated forces [21], and current and voltage sensors provide information to compute the power.

### 2.3. Materials

While previous studies tested various properties of the fin design, including fin shape, fin material stiffness [21], and fin configuration [13,22], we focus this paper on a single fin design parameter: material stiffness. Three fins of different stiffness are introduced in Table 3. Everything apart from the material choice remaining the same, including the shape and the process of fin fabrication and experimental stiffness characterization, is explained in Sampath et al. [23]. The nylon fin is the most rigid material choice, with the two other materials cast into the fin shape being mixtures of polydimethylsiloxane (PDMS). The ratio between the silicone elastomer curing agent and the silicone elastomer base determines the softness (as measured by the elastic modulus) of the polymer, where a higher base indicates higher softness.

### 2.4. Data Collection

The parameter space for the kinematics variables is large. Experimental data are first constrained to parameter values that are physically achievable given an operating frequency. We first limit the amplitude to +/− 90 degrees, as in both the experimental setup and an onboarded vehicle, as sweeping more than 180 degrees is not possible. At higher frequencies, the fins are physically unable to reach certain stroke and pitch amplitudes based on the frequency, as the duration of the gait is shorter. This creates an inverse relationship between the frequency, stroke, and pitch amplitudes. Equation (1) defines the maximum achievable strokes and pitches with respect to frequency based on the given mechanical constraints for the flapping fins:

(1) $$\mathrm{Attainable}\;\mathrm{gaits}:\left\{\begin{array}{c} \Phi =68*f^{-1.4}\\ \Theta =50*f^{-1}. \end{array}\right.$$

With this equation, we collected data that cover the entire scope of the achievable kinematics range at each frequency. In total, 864 unique gait combinations listed in Table 4 were tested, as they covered the full range of gaits while taking a reasonable amount of time to collect data. Each cycle took approximately 2 min, making the total time for all three materials upwards of 80 h. For each fin gait, ten flap cycles were run. Only the five middle cycles were used for analysis to account for discrepancies when the actuator started and ended the cycle motions. Recorded sensor data were converted into final values using a MATLAB post-processing script. Data were collected in a zero velocity flow condition, which previous research demonstrated is important for low-speed maneuvering to station-keep and offset buoyancy [21].

This process was replicated for all three fin material designs. Using the experimental data for each trial, we computed the total power consumption of the fin with Equation (2).

(2) $$P=I_{\phi }*V+I_{\theta }*V.$$

**Table 4:** Gait combinations.

Parameter	Values
Stroke Amplitude (°)	0, 15, 25, 32.5, 40, 55
Pitch Amplitude (°)	0, 15, 25, 32, 38, 55
Frequency (Hz)	0.75, 1.00, 1.25, 1.50, 1.75, 2.00
Stroke Pitch Offset (°)	−22.5, 0, 22.5, 45
Voltage (V)	Constant at 4.98 V

## 3. The Forward Model

With the data collected from the experimental trails, forward models were constructed to predict the stroke-averaged fin thrust and the stroke-averaged power consumption for a flapping cycle from the static gait information. While reduced-order analytic models can produce fast predictions, they struggle to maintain accuracy when generalized beyond a small parameter space [24]. A model supporting a higher-order input space will allow future forward gait-to-propulsion models to incorporate fluid dynamics-related parameters such as flow speed as well as multi-fin kinematic parameters such as the flapping phase offset between front and rear fins.

Neural network surrogate models support higher degree input spaces, and prior flapping fin propulsion research on fin design has developed neural network surrogate models for thrust prediction [25,26]. Both works demonstrate that time series models can predict the time history of thrust generation for a flapping cycle. Therefore, we implemented and compared the accuracy and runtime performance of various reduced-order and high-order approaches using the inputs in Table 1. LSTM networks process sequential data by generating an output at each time step and using information from past outputs to inform subsequent results. Compared to traditional recurrent neural networks, LSTM networks include a cell state to retain a long-term memory accumulated from multiple past time steps that influence the next output.

We developed high-order models using the following criteria:

1. A baseline model that can accurately take in kinematics data (frequency, stroke amplitude, pitch amplitude, and offset) to output predicted power;
2. A capability to take in static information such as material stiffness to use different models to maximize the accuracy and usefulness of the integrated model;
3. A run-time speed of 100 forward passes per second at minimum, with computational power not exceeding the capacity of a Raspberry Pi. We chose 100 as this would allow the system to invoke 50 calls in under half a second, allowing for real-time optimization.

Separate forward models have been developed for various design-related parameters such as material, design, rigidity, and tandem fin spacing. Since these installed parameters are static and will not be replaced during missions, a forward model is more accurate for cycle-by-cycle calculations and only needs to load what is relevant to the mission without wasting excess computational power. Forward models are trained on dynamic gait information such as frequency, stroke angle, pitch angle, angle offset, and tandem phasing.

### 3.1. Model Development and Selection

We examined six approaches to model thrust and power; two were reduced-order polynomial models, and four utilized high-order deep learning approaches.

**Figure 4:**
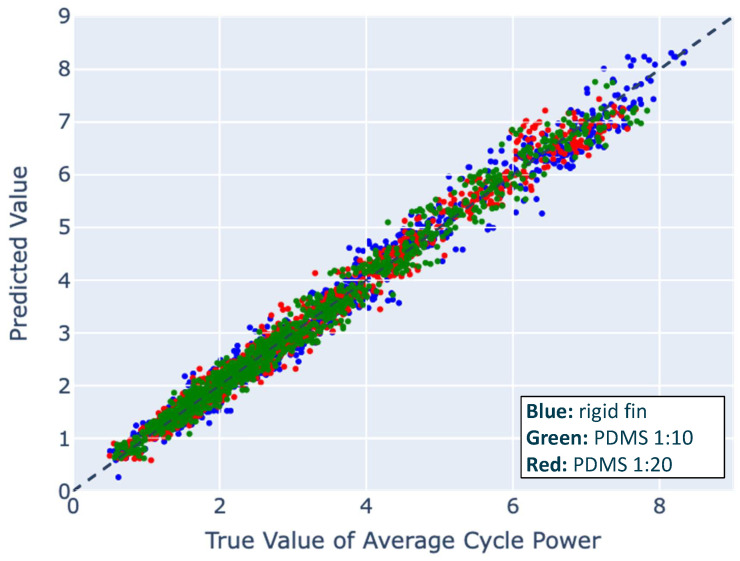
The performance of all three reduced-order quartic polynomial models on synthetic data, comparing the predicted power value to the true power value. Colors indicate the material, with blue (rigid), green (1:10), and red (1:20).

We explored linear models from degrees one to five and found that the quartic model best fits the data. Using the linear model as a baseline, the model performed the fastest with an error averaged across all three models and materials of 0.3891 W and 0.1638 N. As the true value and predicted values appeared to have an exponential relationship in the linear model, we tested a quartic polynomial, which produced better results (Figure 4) with an error averaged across all three models and materials of 0.1815 W and 0.0911 N. Out of the three data sets, the PDMS 1:10 fins fit the best to the quartic regression.

To explore high-order approaches, we implemented multi-layer perception regression (MLP), a convolutional neural network (CNN), and a dense neural network (DNN) that each use the static, kinematic values in Table 1 to directly predict average thrust and power. In addition, a long short term memory (LSTM) model utilized time series in addition to static values. Of these high-order approaches that did not utilize time series, DNNs, which consist of layers of nodes such that each node in layer *l* is connected to every node in layer l−1, performed the best. Overall, deep learning approaches increased accuracy while operating at a speed fast enough for model integration. Table 5 highlights the benchmarked results for various forward model approaches. Each error was averaged across all three individual models, each trained on one individual material. Power ranged from 0 to 9.3 W, and propulsion ranged from −1.2 to 1.2 N.

Out of all six model approaches, the LSTM had the best performance but was also the most time-consuming. However, although the LSTM approached 100 computations/second on a Raspberry Pi Model 4B, which we defined as our limit for model selection, it still met the benchmark and was selected for implementation over the other models due to its superior accuracy. An additional benefit of the LSTM is that it can utilize the dynamic time series inputs to accurately interpolate between gaps of data, which is useful for understanding how the whole gait space behaves for various movements.

### 3.2. LSTM Results

When trained on all experimental gaits, the thrust LSTM reached an average error of 0.0076 N and the power LSTM reached an average error of 0.0072 W. The most visible shortfall of the power LSTM model was its inability to grasp the time history of power consumption or thrust at certain gaits. Each cycle was vastly different at a low flapping fin frequency combined with a low stroke angle due to random actuator noise creating jolts in the time history that would otherwise be negligible at higher frequency gaits. Even still, it was able to predict the average time history of many cycles, and for the purpose of interpolating the final numerical power consumption or propulsion generated by any given gait, this is not a setback.

After selecting the LSTM model, we trained an LSTM model for each fin material and both thrust and power to 1000 epochs for modeling both power and thrust predictions, tuning each model’s parameters, including the dense layer units, dropout, batch size, learning rate, and points per cycle. The statistics for the LSTM models are found in Table 6.

**Figure 5:**
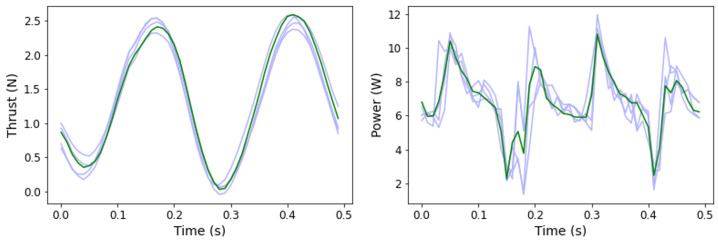
Two sample experimental (blue) and predicted (green) thrust/power time histories for two interpolated kinematics. The left graph involves interpolation to an unseen stroke and pitch angle, while the right graph involves interpolation to an unseen flap frequency and stroke pitch offset.

To test LSTM gait interpolation, a holdout set of gaits was excluded from training. The holdout set consisted of all experimental gaits fulfilling one or more of the following criteria: a flap frequency of 1.25 Hz, a stroke-pitch offset of 0, or a stroke or pitch amplitude of 25°. The accuracies of the LSTM without the holdout sets are shown in Table 7. The limited LSTMs successfully interpolated kinematics for the excluded gaits with a mean average thrust error of 0.0344 N and mean average power error of 0.0853 W. The worst performing subset of excluded gaits, i.e., gaits with a stroke pitch offset of 0, still obtained a mean average thrust error of 0.0374 N. As the LSTM embeds an understanding of how thrust and power change over the course of a flapping cycle, these models capture the peak and troughs of the thrust and power time histories, as shown in Figure 5, for two particular sets of fin kinematics, which were randomly sampled among all of the possible fin kinematics and indicative of the larger set. This understanding of the time histories offers an explanation for the high-accuracy LSTM average predictions for interpolated kinematics.

**Figure 6:**
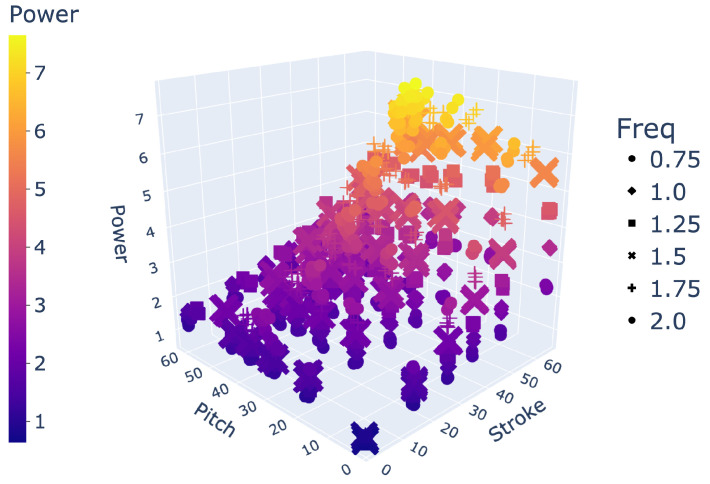
Space of 864 gaits.

**Figure 7:**
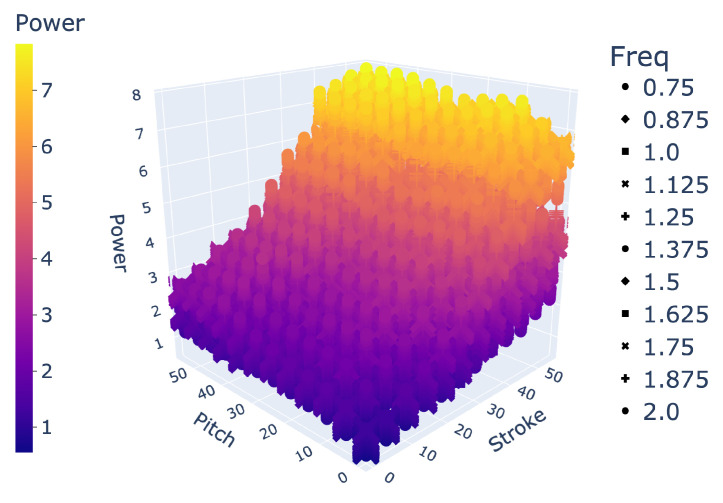
Space of 20,591 gaits.

**Figure 8:**
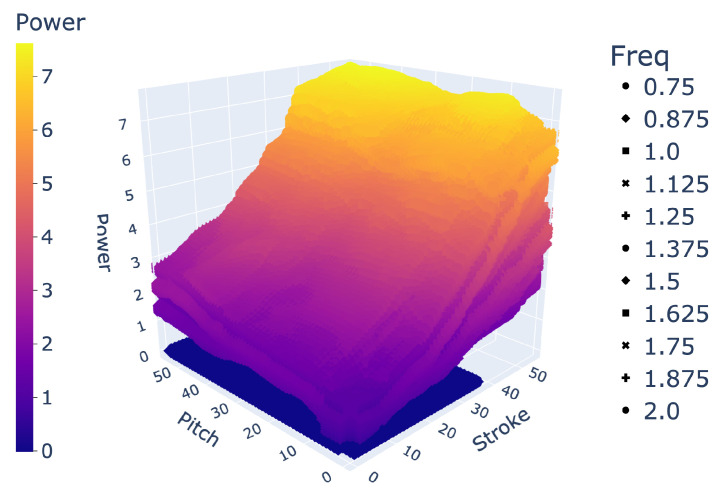
Space of 400k+ gaits.

In demonstrating the ability of an LSTM to interpolate between large holdout sets in data, we showed its effectiveness at filling large gaps in data. For example, we can implement an LSTM to transform our experimentally collected dataset of 864 unique gait combinations to any desired resolution as a higher density array, as visualized in Figure 6. Arrays of 20,591 gaits and over 400,000 gaits, as predicted by an LSTM, are shown in Figure 7 and Figure 8, respectively.

In total, we generated interpolations within the constraints given by the collected data outlined in Equation (1). We interpolated data for every stroke and pitch combination from 0 to 55 degrees with 1 degree increments, frequency from 0.75 to 2 Hz with 0.125 Hz increments, and SPO from −22.5 to 45 degrees with 5.625 degree increments. In total, 435,600 interpolations were calculated for each data set. These interpolations filled gaps of data, creating better insight into how different gait patterns and materials behave for various of-interest combinations, such as at very low frequencies and flapping angles or high frequencies and high angle offsets.

To verify the accuracy of the interpolated high-density arrays, we trained six separate LSTM models for thrust/power on each fin material. In total, 40% of the original training data were withheld, with 20% serving as a test set and 20% serving as a holdout set being intentionally withheld to evaluate the accuracy of interpolations. The resultant errors are within one decimal place of the errors in Table 6, indicating that although there was an increase in the error of the prediction, the predicted thrust and power remained very accurate regardless.

### 3.3. Model Inference Results

To measure the propulsive efficiency of a vehicle or thruster, traditionally, thrust is multiplied by vehicle velocity and divided by power input. Because our thrust results were achieved at zero freestream flow to understand force production near hover and low speed operations, we created a dimensionless figure of merit (FOM) as a surrogate for propulsive efficiency.

To compute the FOM η, the average thrust over a cycle was multiplied by a characteristic velocity scale and divided by the flapping cycle power input, as displayed in Equation (3). This non-dimensional value allowed us to compare across different gaits and inflow conditions. For vehicle integration purposes, this allowed for the return of a pure value or a percentage compared to the highest recorded gait FOM for a loaded design.

(3) $$\eta =\frac{F_{avg}*v}{P_{avg}}.$$

To allow for better comparison across different stroke frequencies and amplitudes in later tests, a universal FOM utilized the average fin tip speed as the characteristic velocity, as shown in Equation (4), where Φ is the stroke amplitude, $r_{tip}=0.18125$ m is the distance from the rotation axis to the fin tip, and *f* is the flapping frequency.

(4) $$v=2\pi *\left(\frac{4*\Phi }{360}\right)*r_{tip}*f.$$

An FOM can also be applied to study different objectives. While we studied the specific propulsive efficiency of thrust, the FOM can isolate how efficiency changes with the individual stroke and pitch actuators or examine the side and lift forces.

**Figure 9:**
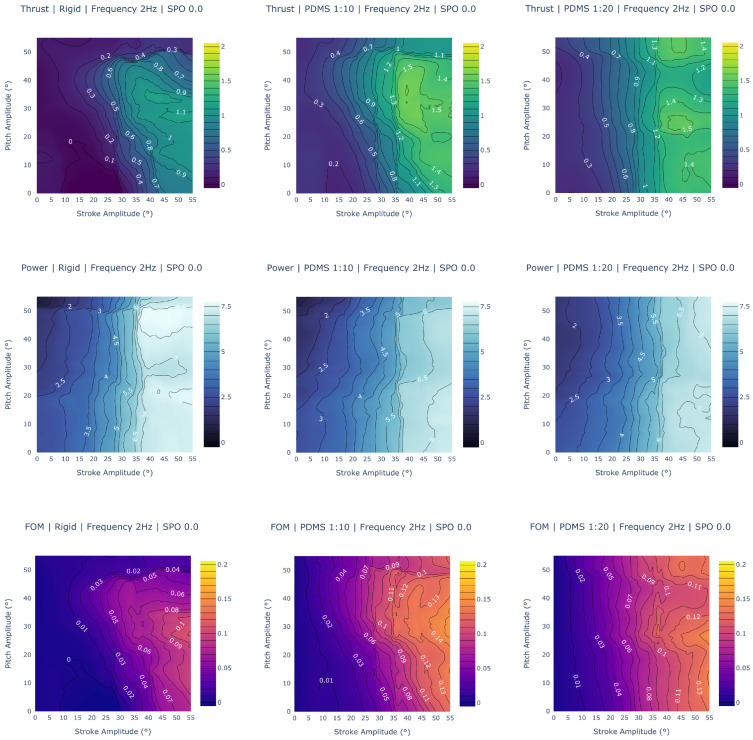
Thrust, power, and figure of merit contours. The columns are different data sets (rigid, PDMS 1:10, and PDMS 1:20) while the rows graph different gait results (thrust, power, and FOM value). The PDMS 1:10 fin generates the highest possible thrust and is higher overall in more gaits; the rigid fin is significantly worse at thrust generation than either of the PDMS fins. The PDMS 1:10 and 1:20 fins are comparable in power consumption but differ in trends at higher stroke and pitch combinations; the rigid fin consumes significantly more power. The PDMS 1:10 fin has the largest FOM values, with the PDMS 1:20 fin following. The rigid fin is significantly worse in all 3 metrics.

To gain a better understanding of how the figure of merit correlates to the thrust force and power consumption, we created a grid of contours for one gait, shown in Figure 9. Here, the stroke and pitch range for a frequency of 2 Hz and stroke-pitch offset of 0° is depicted. The grid contains columns with the three material data sets, and each row graphs part of the figure of merit equation: thrust, power, and FOM, in that order.

### 3.4. Discussion and Analysis of Fin Performance

**Figure 10:**
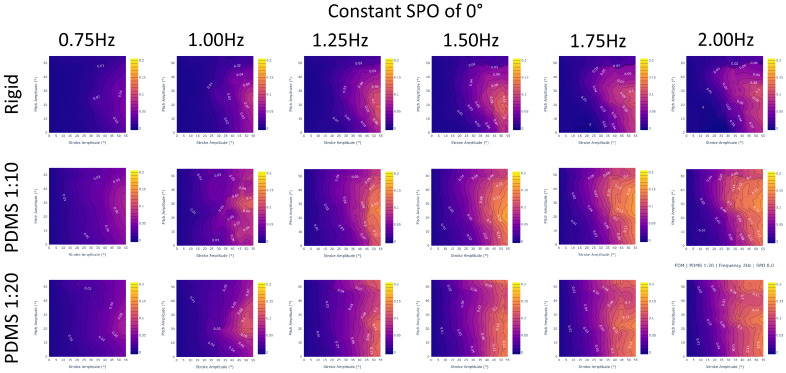
Figure of merit results for a constant stroke–pitch offset of 0 degrees and a varying frequency incrementally increasing from left to right. The columns graph all interpolated frequency values with 0.25 Hz increments while the rows are different data sets (rigid, PDMS 1:10, and PDMS 1:20). We verify that PDMS 1:10 is the best performing across all frequencies with higher FOM values and averages compared to the PDMS 1:20 or rigid fins. Increasing the frequency will improve a gait’s FOM regardless of fin design.

**Figure 11:**
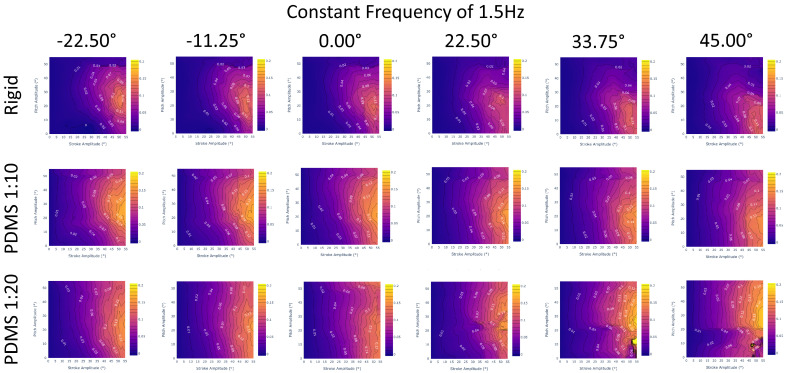
Figure of merit results for a constant frequency of 1.5 Hz and a varying stroke–pitch offset incrementally increasing from left to right. The columns graph all interpolated SPO values with 11.25° increments while the rows are different data sets (rigid, PDMS 1:10, and PDMS 1:20). We verify that the PDMS 1:10 fin is the best performing across all SPO values with higher FOM values and averages compared to the PDMS 1:20 or rigid fins. With the exception of 22.5–45° for PDMS 1:20, a more negative SPO will slightly improve a gait’s FOM regardless of fin design.

To analyze the fin performance, we selected various random slices of the possible parameter space, shown in Figure 9, Figure 10 and Figure 11. These figures are comprised of contours that visualize the thrust propulsion, power consumption, and figure of merit for various given movements; these contour plots are comprised of the full range of possible stroke and pitch amplitude combinations with different materials, frequencies, or stroke-pitch offsets tested across each contour.

Figure 9 compares the performance between all three fin materials across each column for a set frequency of 2 Hz and stroke-pitch offset of 0. Beginning with the thrust in the first row, a few trends are immediately apparent. First, the fin design that can generate the largest force is the PDMS 1:10 design, generating a maximum force of around 1.6 N at a 40° stroke amplitude and 30–40° pitch amplitude. Both the other designs trail behind, with the PDMS 1:20 fin being able to generate a thrust of 1.5 N and the rigid fin only managing up to 1.1 N. The PDMS 1:10 fin also has more gaits at higher thrust levels. An observation of the contour reveals that there are more combinations of stroke and pitch that produce higher thrusts when compared to the PDMS 1:20 fin. An analysis of all gaits, including other variable stroke–pitch offsets and frequencies, confirm these findings. The PDMS 1:10 fin’s maximum thrust is 2.1 N, while the PDMS 1:20 fin produces a maximum force of 1.6 N, and the rigid fin produces a maximum force of 1.2 N. The PDMS 1:10 fin has the highest average thrust generation, followed by the PDMS 1:20 fin.

Power consumption, highlighted in the second row, goes in the reverse order. The design that requires the highest wattage is the rigid fin design, requiring 7.6 W for any gait with a stroke amplitude of more than 40°. The PDMS 1:10 and PDMS 1:20 fins are similar, with a maximum power consumption of 7.1 W. However, in cases above 40° stroke and 30° pitch, the PDMS 1:10 fin observed lower wattage consumed at the same gait combination. The PDMS 1:20 fin appears to depend less on the pitch amplitude, with power more dependent on the stroke amplitude. These observations are verified when looking at all gaits. While the PDMS fins have a maximum wattage of around 7.5 W, they occur at much fewer gaits than with the rigid data set.

Another interesting trend is visible when comparing the FOM and thrust charts, which appear almost identical, with only a few differences in their trends. The explanation becomes evident when looking at the contours for power, which have a near-linear trend across stroke amplitude. While pitch amplitude does affect both the PDMS 1:10 and 1:20 designs, the stroke amplitude has the most recognizable and significant effect. Future work will include generating additional figures to verify that this trend exists across all frequencies and stroke–pitch offsets.

This analysis allows us to conclude that the PDMS 1:10 fin design is the most efficient out of all three designs, with the highest thrust generation and figure of merit values. Following in second is the PDMS 1:20 fin, which has the second-largest thrust generation and figure of merit values. These trends are confirmed across the ranges of stroke and pitch (Figure 9) as well as frequency (Figure 10) and SPO (Figure 11). This suggests that the most efficient design that is able to generate the largest thrust may lie between the two and is something of interest for future exploration.

From Figure 10, we observe that across the entire range of frequencies, the PDMS 1:10 outperforms both the rigid and PDMS 1:20 fin designs. Additionally, we observe that regardless of fin design, increasing the frequency will improve the FOM metric, which is demonstrated by the increasing figure of merit when moving from lower frequencies to higher frequencies across the same selection of gaits.

From Figure 11, we observe that across the entire range of stroke–pitch offset, the PDMS 1:10 outperforms both the rigid and PDMS 1:20 fin designs. Additionally, we observe that regardless of fin design, a more negative offset will slightly improve the FOM metric, although the difference is very marginal. At high SPO, the PDMS 1:20 fin design appears to diverge from the expected trends at 22.5° or higher and invites future exploration. In the future, revisiting the model’s training data for high SPO will likely resolve the issue.

We conclude the following from the interpolated data:

1. The best-performing fin design is PDMS 1:10, followed by PDMS 1:20. Both consume similar amounts of power, but PDMS 1:10 fins produce a higher thrust. The rigid fin consumes more power and produces less thrust.
2. The most optimal fin design likely lies in between the PDMS 1:10 and 1:20 fins.
3. The most efficient gait occurs at a high stroke (40–55), centered pitch (20-35), high frequency (2 Hz), and low SPO (−22.5°).

## 4. Discussion

We introduced three materials of varying stiffness, collecting 864 unique gaits that span the physically possible combinations of four parameters that define a gait. These data were used to train and evaluate six different models for thrust and power to interpolate between experimental data with high accuracy: the linear model, quartic polynomial model, convolutional neural network, dense neural network, and long short-term memory model. All six evaluated models accomplished all three criteria we laid out. They were also able to do the following:

- Complete a baseline model that inputs gait parameters to output thrust or power with minimal error;
- Retrain on different designs and maintain similar or better accuracy;
- Run at a speed and size suitable for real-time integration into a control system (>100 computations per second).

These criteria allow for our developed models to contribute noticeable advancements compared to prior CFD models, which are too computationally intensive and slow for real-time deployment in an unmanned system. Our model is fast enough to operate in real-time, and the low computational cost allows for the model to be run in the unmanned system itself. This general framework of using physically-collected gait data to train data-driven surrogate models offers the ability to replace or augment the use of prior CFD simulations and is transferable to other systems where gait characteristics can be easily broken down into various parameters attributed to the propulsive output and power consumption of any given movement.

Out of all six models, we found that the LSTM model was able to produce the most accurate results on the full data set. When we removed specific gaits to test the ability of the model to create interpolations on unseen data, a limited amount of accuracy was sacrificed, although the model was still able to predict the value with low error. On the training data, the more limited thrust LSTM model’s aggregate average was 99.65% with a 0.0076 N error; on a holdout set of unseen data, the thrust LSTM model’s aggregate average was 98.68% with a 0.0344 N error. On the training data, the power LSTM model’s aggregate average was 99.56% with a 0.0408 W error; on a holdout set of unseen data, the more limited power LSTM model’s aggregate average was 99.08% with a 0.0853 W error. This allowed us to demonstrate that the LSTM is able to accurately interpolate unseen and withheld gaits with minimal error, enhancing the resolution of our data.

We used two forward-passing LSTM models to generate kinematic interpolations of gait-to-thrust and gait-to-power predictions with the goal of optimizing the efficiency of gaits and providing a better understanding of material designs and their relation to efficiency. This was carried out through a dimensionless figure of merit that compared the fin efficiency to other flapping systems and evaluated the efficiency of gaits onboard the control system. Using the generated interpolations, we were able to create contours that visualized how efficient a given area of gaits was, creating comparisons between varying frequencies, stroke–pitch offsets, and materials.

With the FOM, we concluded that both PDMS materials were more efficient than the rigid fin, with the PDMS 1:10 fin generating the maximum thrust with the lowest power consumption. We validated this by comparing the average thrust and average FOM across all possible gaits for each fin material. When comparing the average thrust propulsion across all gaits, the rigid material had an average of 0.3407 N thrust, the 1:10 material had an average of 0.6047 N thrust, and the 1:20 material had an average of 0.5963 N thrust. Compared to the rigid design, the 1:10 and 1:20 materials saw a 83.89% and 77.49% increase, respectively. There was only a 1.41% improvement between the 1:10 and 1:20 designs. However, as the 1:20 design compared significantly more power, the 1:10 material saw significant improvements in the average FOM. When comparing the average FOM across all gaits, the rigid material had an average FOM of 0.0886, the 1:10 material had an average FOM of 0.2105, and the 1:20 material had an average FOM of 0.1615. Compared to the rigid design, the 1:10 and 1:20 materials saw a 137.58% and 82.28% increase, respectively. This created a much larger 30.34% improvement between the 1:10 and 1:20 designs.

There were observable trends consistent across all materials. To optimize the efficiency of any given kinematic, we observed that efficiency improved for a kinematic with a higher frequency, higher stroke amplitude, and highly negative stroke–pitch offset. All three criteria contributed to greater efficiency; pitch amplitude had a significant effect on the overall efficiency of the gait. At a constant pitch amplitude of 55°, increasing the frequency by 1.25 Hz from 0.75 Hz raised the FOM by 0.08 on average, and increasing the stroke–pitch offset by 67.50° from −22.50° raised the FOM by 0.03 on average. Meanwhile, raising the pitch amplitude from 0° to 55° increased the FOM anywhere from 0.1 to 0.17, depending on the gait being at a low and high frequency, respectively. The most efficient gait was concluded to be for the PDMS 1:10 data set with a −22.5° stroke–pitch offset and frequency of 2 Hz. This understanding will allow us to design fins that generate a higher thrust and maintain the highest power efficiency, as well as tune an inverse search model to search gaits that are known to be power-efficient or optimal.

We are currently in the deployment stage and collecting experimental data for in-tank testing of the control system to evaluate and, if needed, retrain the forward model by running physical simulations in underwater environments. The results of full simulated trials will be further detailed, with recommendations made to set varying fin materials and test settings/environments.

## 5. Conclusions

This study presented material insights into the effect of stiffness on propulsion and efficiency outcomes. We developed and tested two forward gait-to-thrust and forward gait-to-power models to predict the thrust and power outcomes of various kinematics using six LSTM models with an average error of 0.0076 N and 0.0072 W across all three materials. When trained on a smaller set of data, we validated that the LSTM models are successfully able to interpolate known values withheld from model training with a marginal increase in error. Notably, this paper demonstrated that the forward models trained can capture the parameters with high resolution. This allows for interpolating between known data points and enables thrust and FOM gains through proper materials and kinematics selection permissible because of the model’s ability to interpolate high-resolution data. Using a dimensionless figure of merit, we can concluded that both flexible PDMS 1:10 and 1:20 materials are more efficient and propulsive compared to the rigid fin, with the PDMS 1:10 fin being the most efficient and propulsive overall. Across an average of all gaits, the 1:10 mixture improved the thrust generated from the same gait by approximately 83.89% compared to the rigid fin and the average FOM by approximately 137.58% compared to the rigid fin and 30.34% compared to the 1:20 fin.

## Figures and Tables

**Table 1:** Kinematics gait parameters.

Parameter	Symbol	Description
*Static Kinematics*		
Frequency (Hz)	*f*	Number of flap cycles per second
Stroke Pitch Offset (°)	δ	Phase offset of the pitch cycle to the stroke cycle, calculated as $\frac{1}{16}$th of one cycle
Stroke Amplitude (°)	Φ	Maximum stroke angle over one flap cycle
Pitch Amplitude (°)	Θ	Maximum pitch angle over one flap cycle
*Dynamic Kinematics*		
Stroke Angle (°)	ϕ	Time history of stroke angle
Pitch Angle (°)	θ	Time history of pitch angle

**Table 2:** Control system measurements.

Parameter	Symbol	Description
*XYZ Forces*		
Thrust (N)	*T*	Force generated along stroke axis
Lift (N)	*L*	Force generated perpendicular to both
Side Force (N)	*S*	Force generated along pitch axis
*Power Consumption*		
Stroke Current (A)	$I_{\phi }$	Time history of current draw for the stroke actuator
Pitch Current (A)	$I_{\theta }$	Time history of current draw for the pitch actuator
Voltage (V)	*V*	Voltage of both actuators

**Table 3:** Fin material properties.

Material	Young’s Modulus
Rigid Nylon	1 GPa
Polydimethylsiloxane (PDMS) 1:10	850 kPa
Polydimethylsiloxane (PDMS) 1:20	310 kPa

**Table 5:** Averaged model performances.

Model	Averaged Error (W)	Averaged Error (N)	Runtime
Linear Polynomial	0.3891	0.1638	Low
Quartic Polynomial	0.1815	0.0911	Low
MLP	0.1229	0.0638	Medium
CNN	0.0907	0.0572	Medium
DNN	0.0429	0.0364	Medium
LSTM	0.0072	0.0076	High

**Table 6:** Average error for LSTM models.

Full Models	Rigid	PDMS 1:10	PDMS 1:20
Power (W)	0.0236	0.0720	0.0268
Thrust (N)	0.0002	0.0186	0.0041

**Table 7:** Average error for LSTM models excluding holdouts.

Interpolations	Rigid	PDMS 1:10	PDMS 1:20
Power (W)	0.0574	0.1296	0.0689
Thrust (N)	0.0057	0.0759	0.0216

## Data Availability

Raw data were collected at the Naval Research Laboratory. Derived data supporting the findings of this study are available from the corresponding author K.V. on request.
